# High Prevalence of Systemic Autoimmune Diseases in Patients with Menière's Disease

**DOI:** 10.1371/journal.pone.0026759

**Published:** 2011-10-28

**Authors:** Irene Gazquez, Andres Soto-Varela, Ismael Aran, Sofia Santos, Angel Batuecas, Gabriel Trinidad, Herminio Perez-Garrigues, Carlos Gonzalez-Oller, Lourdes Acosta, Jose A. Lopez-Escamez

**Affiliations:** 1 Genyo, Centro de Genómica e Investigación Oncológica, Pfizer/Universidad de Granada/Junta de Andalucia, Granada, Spain; 2 Division Otoneurology, Department of Otorhinolaryngology, Complejo Hospitalario Universitario Santiago de Compostela, Santiago de Compostela, Spain; 3 Department of Otolaryngology, Complejo Hospitalario de Pontevedra, Pontevedra, Spain; 4 Department Otolaryngology, Hospital Univesitario Salamanca, Salamanaca, Spain; 5 Department Otolaryngology, Hospital Universitario de Badajoz, Badajoz, Spain; 6 Division Otoneurology, Department of Otorhinolaryngology, Hospital La Fe, Valencia, Spain; 7 Otology & Neurotology Group, CTS495, Department of Biotechnology, Hospital de Poniente, El Ejido, Almeria, Spain; 8 Otology & Neurotology Group, CTS495, Department of Otolaryngology, Hospital de Poniente, El Ejido, Almería, Spain; Yale Medical School, United States of America

## Abstract

**Background:**

Autoimmunity appears to be associated with the pathophysiology of Meniere's disease (MD), an inner ear disorder characterized by episodes of vertigo associated with hearing loss and tinnitus. However, the prevalence of autoimmune diseases (AD) in patients with MD has not been studied in individuals with uni or bilateral sensorineural hearing loss (SNHL).

**Methods and Findings:**

We estimated the prevalence of AD in 690 outpatients with MD with uni or bilateral SNHL from otoneurology clinics at six tertiary referral hospitals by using clinica criteria and an immune panel (lymphocyte populations, antinuclear antibodies, C3, C4 and proinflammatory cytokines TNFα, INFγ). The observed prevalence of rheumatoid arthritis (RA), systemic lupus erythematosus (SLE) and ankylosing spondylitis (AS) was higher than expected for the general population (1.39 for RA, 0.87 for SLE and 0.70 for AS, respectively). Systemic AD were more frequently observed in patients with MD and diagnostic criteria for migraine than cases with MD and tension-type headache (p = 0.007). There were clinical differences between patients with uni or bilateral SNHL, but no differences were found in the immune profile. Multiple linear regression showed that changes in lymphocytes subpopulations were associated with hearing loss and persistence of vertigo, suggesting a role for the immune response in MD.

**Conclusions:**

Despite some limitations, MD displays an elevated prevalence of systemic AD such as RA, SLE and AS. This finding, which suggests an autoimmune background in a subset of patients with MD, has important implications for the treatment of MD.

## Introduction

Meniere's disease (MD) is a chronic disorder affecting the inner ear characterized by fluctuating sensorineural hearing loss (SNHL), episodes of vertigo lasting from 20 minutes to hours, tinnitus, and aural fullness [1]. Phenotypic heterogeneity is observed in patients with MD and it is difficult to define the outcome in early stages of the disease. Although the frequency of the attacks of vertigo is usually greater in the first few years of the disease, and it diminished at advances stages of MD [2], [3], balance problems persist along the disease and may become severe if patients develop a bilateral vestibular hypofunction.

Hearing loss can be observed in one or both ears. The prevalence of bilaterality is around 25–40%, being dependent upon the follow-up period, and the risk of developing bilateral MD for a patient with unilateral disease has been estimated 14% [4]. Usually, SNHL starts in one ear and it can appear in the second ear after a mean interval of 7 years (range: 2 months −27 years) [4], and bilateral SNHL has a significant impact in health-related quality of life in MD [5].

Autoimmune mechanisms appear to be associated with the pathophysiology of MD [6], [7]. The evidences that support this hypothesis include: the association with alleles of DRB1 gene of the MHC reported in different populations [8], [9]; the finding of elevated levels of autoantibodies or circulating immune complexes (CIC) in the serum of some patients [7], [10], [11]; the experimental induction of hydrops by injection of antigens or monoclonal antibodies in the guinea pig [12] and the association with a functional variant of LYP, a lymphoid protein phosphatase, which inhibits T cell receptors response in patients with bilateral ear involvement [13]. Different autoantibodies such as antinuclear antibodies (ANA), antiphospholipid antibodies and antibodies against a 68 kD protein have been studied in small series of patients with MD, showing conflicting results [6], [7], [14]–[17]. However, the prevalence of autoimmune disease (AD) in patients with MD or the role of lymphocyte subpopulations and cytokines such as TNFα and INFγ has seldom been investigated in patients with MD [17].

The aim of this study is to estimate the prevalence of AD in patients with MD and to evaluate an immune panel among patients wih MD and unilateral or bilateral SNHL.

## Methods

### Subjects

A case series of 690 adult patients (217 bilateral, 473 unilateral disease) with a clinical diagnosis of definite MD according to the diagnostic scale of the American Academy of Otolaryngology- Head and Neck Surgery (AAO-HNS) were included from October 2007 to May 2011 [18]. We selected patients with bilateral SNHL in a higher percentage than it is observed in most clinics to reach enough number of patients to be compared with unilateral MD. All were outpatients regularly followed up at the Otoneurology Clinics at six tertiary referral centers that recruited patients for this study. The information was obtained by means of a standardized, structured clinical database designed for follow-up in patients with MD [19]. When patients agreed to participate in the study, they were informed that they must provide information about the number and duration of episodes of vertigo, headache and hearing loss they had experienced in the last six months. The interview was oriented to identify the type of headache and if the patient reported symptoms suggesting an autoimmune condition.

All patients underwent complete otolaryngologic examination and audiologic evaluation with pure-tone audiometry and tympanometry. A neurotologic examination including nystagmus in the primary position, gaze-evoked and head-shaking nystagmus and standard bithermal caloric test was also performed. Hearing staging for each patient with definite MD was defined as the mean of four-tone average of 0.5, 1, 2 and 3 kHz according to the AAO-HNS criteria: stage 1, ≤25 dB; stage 2, 26–40 dB; stage 3, 41–70 dB; 4, stage 4, >75 db [18]. Patients with bilateral SNHL were considered to have metachronic SNHL if a period of time >1month to develop SNHL between the first and the second ear was observed. Individuals with simultaneous SNHL in both ears were considered to have synchronic SNHL. Episodes of vertigo were characterized by their frequency and duration as previously described [16], and episodes of vertigo lasting less than 20 minutes or the sensation of instability usually observed in these patients were not considered in this study. The protocol of diagnosis included an examination by magnetic resonance imaging of the brain to exclude other possible causes of neurotological symptoms.

### Ethics

The subject's written informed consent was obtained to participate in the study according to the Helsinki guidelines and the Hospital de Poniente Institutional Review Board approved the study. The clinical features of a subset of 89 patients with MD from Almeria in our series were previously reported [9].

### Immunoserological assessment

Antinuclear antibodies (ANA) were detected on Hep-2 cells by indirect immunofluorescence method. Titers of 1/160 were considered positive. We also used a qualitative enzyme-linked immunosorbent assay (ELISA) for the detection of ANAs (DIASTAT). It detects ANAs against Sm, Sm/RNP, Ro (SS-A), La (SS-B), Scl-70, Jo-1, dsDNA histone and centromere antigens. Serum concentrations of extractable nuclear antigens (ENAs) were detected by EliA Symphony (Pharmacia Diagnostics, Freiburg, Germany), an ENA screening which detects the following autoantibodies: SSA/Ro, SSB/La, U1RNP (A, C), RNP70, Scl-70, JO-1, centromere B and Sm. Also, anti-dsDNA were measured by EliA dsDNA (Pharmacia Diagnostics, Freiburg, Germany).

Serum complement proteins C3 and C4 and immunoglobulins (IgG, IgM, IgA) were measured by an immunoturbidimetric assay on an Olympus AU5400 analyzer.

A C1q binding ELISA kit (HYCOR, Agilent Technologies, Amstelveen, The Netherlands) was used to determine Circulating Immune Complexes (CIC) concentrations in patients with MD in a Hytec 288 Plus System (HYCOR). Values higher than +3 SD of the mean normal cutoff point were considered positive.

Peripheral lymphocyte populations were characterized by an EPICS XL-MLC flow cytometer (Coulter). Fluorescent dyes used were CD3-PC5/CD4-FITC/CD8-PE.

### Determination of serum soluble cytokines

Serum interferon-γ (IFN-γ) and tumor necrosis factor-α (TNF-α) were measured by an ELISA kit (Biosource TM, Nivelles, Belgium) using an ELISA plate reader (Menarini Diagnostics) according to manufacturer's instructions. The intraassay and interassay coefficients of variation, respectively, were 1.8% and 2.3% for IFN-γ, 0.9% and 1.6% for TNF-α. All analytical determinations were performed in the quiescent phase (at least 4 weeks since the last episode of vertigo).

### Main outcome measurements

The clinical records of all patients were reviewed to retrieve additional information not included in the clinical database. Some patients were visited several times to obtain all clinical and analytical variables of the protocol. The following variables were assessed: sociodemographics (sex, age), clinical (age of onset, uni/bilateral disease, ear affected, hearing loss at diagnosis, AAO-HNS hearing stage, class (frequency of vertigo during the last six months), Tumarkin crisis and functional scale, duration of attacks, time- course of the disease, time since last attack) and associated factors (headache, type of headache, history of AD, smoking and coffee drinking). Diagnosis of AD was performed by a rheumatologist or Internal Medicine specialist, according to the criteria defined by the American College of Rheumatology (http://mail3.rheumatology.org/practice/index.asp). Diagnosis of migraine and tension-type headache was performed according to the International Classification of Headache Disorders [20].

### Exclusion criteria

We excluded patients with vestibular migraine, benign paroxysmal positional vertigo, vestibular neuritis, head trauma, ear surgery, recurrent infection of the middle ear, acoustic schwannoma and any known cause mimicking MD, according to the diagnostic scale of the AAO-HNS [18].

### Statistical analysis

Data are shown as means with standard deviations (SD). Qualitative variables were compared between unilateral and bilateral MD by using Pearson Chi-square with Yates' continuum correction; Fisher's test was calculated when the number of cases per cell was <5. Quantitative variables (age, time-course and time since last attack) were compared by unpaired student's T test. The time-course of hearing stage, episodes of vertigo and Tumarkin crisis were detemined according to the Kaplan-Meier method, and survival curves were compared using the log-rank test.

Bivariate regression analysis was used to search for association between clinical and analytical factors and hearing loss and frequency of vertigo. Using pure tone average hearing loss and the frecuency of vertigo in the last six months as dependent variables, we carried out several multiple linear regression models (forward method) to investigate the factors associated with hearing loss and the frequency of vertigo in patients with MD. Statistical significance was accepted at p<0.05.

## Results

### Clinical differences in patients with uni and bilateral SNHL

The patients in this series showed no differences in age at inclusion or the age of onset of disease (Table 1). However, the percentage of women included in each center was different (range 42–72, p = 0.001). Moreover, although the percentage of patients with bilateral SNHL was different in each center (range 10–47, p = 2.1×10^−8^), they do not represent the real percentage of uni or bilateral MD observed in each clinics.

**Table 1:** Clinical variables of 690 patients with MD according to the center of origin.

CENTERS	Age†	Age of onset†	Gender (% women)	Bilateral sensorineural hearing loss	Follow-up period >10 years (%)
**Almeria (n = 150)**	56.6±12.6	46.6±12.6	61.3	42.7	51 (34)
**Badajoz (n = 104)**	53.4±13.6	46.3±15.2	51.0	9.6	33 (32)
**Pontevedra (n = 127)**	54.4±12.5	46.3±12.4	71.7	30.7	40 (31)
**Salamanca (n = 99)**	53.9±12.5	45.9±12.3	60.6	47.5	29 (29)
**Santiago (n = 155)**	54.0±13.8	45.5±14.4	54.2	41.9	55 (35)
**Valencia (n = 55)**	59.4±13.5	44.1±15.1	41.8	41.9	36 (65)
**P value**	0.042	0.884	0.001	2.1×10^−8^	

† Mean ± Standard deviation.

We compared the clinical phenotype in patients with unilateral and bilateral SNHL with at least 5 years since the onset of symptoms (n = 466, Table 2). Hearing loss at diagnosis was significantly worst in patients with bilateral SNHL when they were compared with unilateral MD (p = 2.9×10^−6^). Migraine was not found more common in patients with uni or bilateral SNHL (p = 0.88). In addition, no differences were found in the frequency of reported rheumatic diseases, smoking or coffee consumption between patients with unilateral or bilateral SNHL. Hearing stage and functional level were worst in individuals with bilateral hearing loss (p = 5.2×10^−8^ and 0.004, respectively).

**Table 2:** Clinical phenotype in 466 patients with MD with at least 5 years since the onset of the disease.

VARIABLES	BILATERAL (n = 191)	UNILATERAL (n = 275)	P value
Age of onset (SD)	45 (14)	44 (12)	0,64
Gender (% women)	55.0	57.4	0.60
Affected ear (%)	40.3	Left, 32.0Right 27.7	
Hearing loss at diagnosis*, mean (SD)	59.3 (15.7)	49.9 (19.3)	2.9×10^−6^
Headache, n (%)	65 (38)	77(31)	0.17
Migraine, n (%)	20 (10)	30 (11)	0.88
Rheumatoid history, n (%)	28 (15)	35 (13)	0.83
Smoking, n (%)	37 (19)	62 (23)	0.11
Coffee drinking n (%)	39 (20)	54 (20)	0.92
Hearing stage, n (%)	Worst ear	Affected ear	
1	3 (2)	25 (10)	
2	37 (22)	84 (34)	
3	85 (50)	106 (43)	
4	46 (27)	30 (12)	
Time-course (years) mean +/− SD	13±8	11±7	0.002
Hearing stage**, mean (SD)	3.0 (0.7)	2.6 (0.8)	5.2×10^−8^
Class, n (%)			
A	66 (35)	120 (44)	0.05
B or C	125 (65)	155 (56)	
Tumarkin crisis n (%)	27 (25)	31 (19)	0.23
Functional scale, n (%)			
1	32 (18)	47 (19)	0.004
2	44 (25)	81 (33)	
3	37 (21)	58 (23)	
4	27 (16)	42 (17)	
5	22 (13)	6 (2)	
6	4 (2)	3 (1)	

* Pure tone avarage (PTA, hearing loss at diagnosis) and time-course were compared by unpaired Student's t test. The rest of qualitative variables were compared by Chi-squared test.

** Hearing stage calculated for the worst ear in bilateral MD.

**Figure 1:**
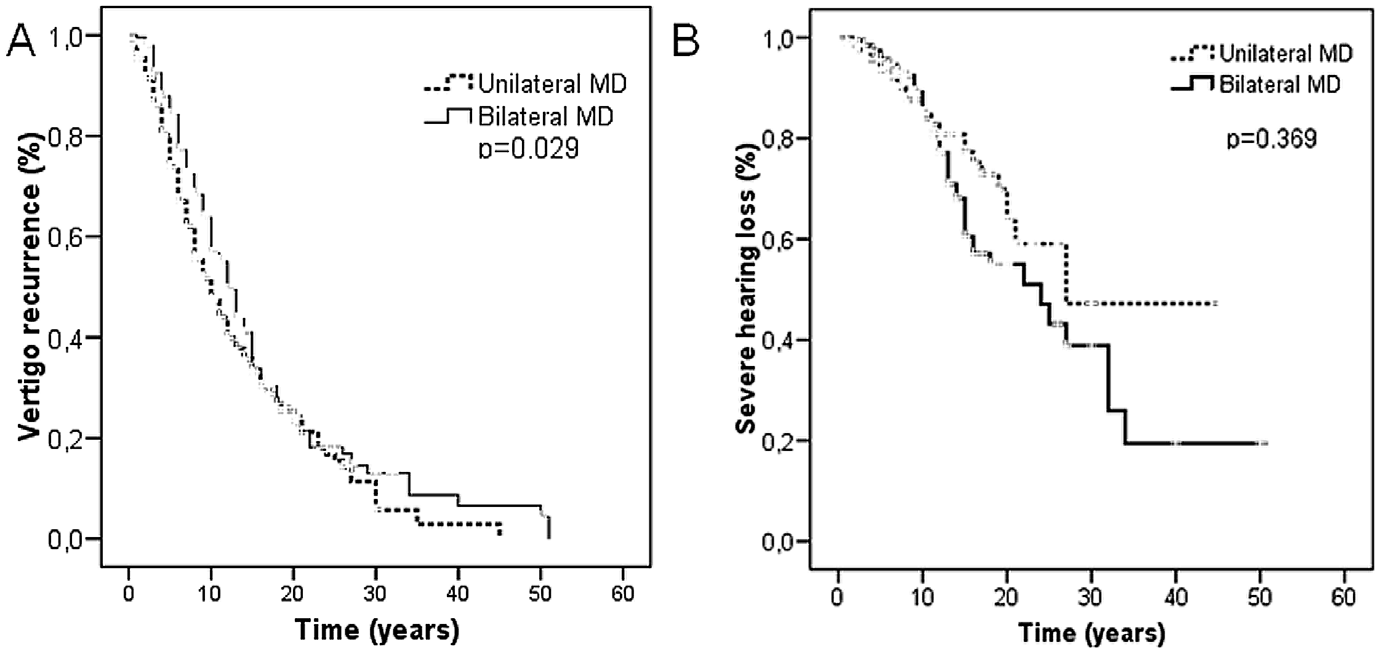
Median duration of recurrent vertigo was longer in patients with bilateral MD (A, log-rank test, p = 0.03). Time to reach hearing stage 4 was used to compare hearing loss progression among patients with uni or bilateral MD (B, log-rank test, p = 0.36).

The vertigo status was assessed in a cohort of 673 patients (444 unilateral and 229 bilateral). The median duration of recurrent vertigo was 10 years (95% confidence interval, 8.56–11.44) in patients with unilateral MD; however, the median duration of recurrent vertigo in patients with bilateral MD was significantly longer (12 years (10.4–13.6), log-rank test, p = 0.03; Fig. 1A). The time-course of hearing stage was also examined in 611 patients (403 unilateral and 208 bilateral). The median of years to reach stage 4 (>75 dB) was 27 and 24 years in patients with unilateral and bilateral SNHL, respectively (log-rank test, p = 0.36; Fig. 1B).

### High prevalence of autoimmune diseases

AD was assessed in a subset of 575 patients in our series (Table 3). Eight patients had rheumatoid arthritis (RA), five were diagnosed of systemic lupus erythematosus (SLE) and four subjects had ankylosing spondylitis (AS). The estimated prevalence of these systemic AD in our series of MD was 1.39 for RA, 0.87 for SLE and 0.70 for AS. The rest of immune conditions were observed with the same frequency that expected in the general population in Caucasians. The overall estimated prevalence of ADs did not differ between patients with uni or bilateral SNHL (p = 0.17). However, AD were most commonly observed among patients with migraine than subjects with tension-type headache (p = 0.007). There were 72 patients with migraine and MD in our cohort, and 19 of them (26%) of had a diagnosis of an AD. However, only 10 of 98 (10%) patients with tension-type headache has AD.

**Table 3:** Autoimmune diseases diagnosis found among 575 patients with MD†.

Disease	Unilateral (N = 378)	Bilateral (N = 197)	TOTAL	Prevalence
Rheumatoid arthritis	5	3	8	**1,39**
Psoriasic arthritis	1	0	1	0,17
Oligoarthritis	1	0	1	0,17
Ankylosing spondilytis	0	4	4	**0,70**
Paget disease	1	0	1	0,17
Rheumatic polymialgia	1	1	2	0,35
Systemic lupus erythematosus	4	1	5	**0,87**
Sjogren Syndrome	2	1	3	0,52
Autoimmune hypothyroidism	7	0	7	1,22
Graves disease	2	2	4	0,70
Autoimmune thyroiditis	2	0	2	0,35
Type I diabetes	1	1	2	0,35
Autoimmune thrombocytopenia	1	0	1	0,17
Primary liver cirrosis	1	0	1	0,17
Autoimmune hepatitis	0	1	1	0,17
Ulcerative colitis	2	1	3	0,52
Rheumatoid fever	2	0	2	0,35
Neumonitis	1	0	1	0,17
Sarcoidosis	0	1	1	0,17
Psoriasis	1	3	4	0,70
Undetermined	4	5	9	1,57
Overall, N	39	24	63	
Prevalence	10,29	12,18	10,95	

† Bold values are higher than expected in the general population.

### Immunological profile in patients with MD

Immunological testing did not found differences for ANA, C3, C4, CIC, IgG, IgM, IgA or lymphocytes population between patients with MD and uni or bilateral SNHL (Table 4). Serum levels of TNFα and INFγ were not different from controls in either unilateral or bilateral MD (Table 5).

**Table 4:** Comparison of standard immunological variables between patients with unilateral and bilateral MD.

	Total	Bilateral	Unilateral	P value
ANA IIF 1/160 (%)	30/400 (7)	12/153 (8)	18/247(7)	0.85
ANA ELISA (%)	7/71 (10)	3/33 (9)	4/38 (10)	1.00
ENA (%)	14/315 (4)	8/135 (6)	6/180 (3)	0.28
Anti dsDNA	4/116 (3)	2/46 (4)	2/70 (3)	0.65
C3 increase (%)	42/437 (10)	20/171 (12)	22/266 (8)	0.49
C4 increase (%)	55/437 (13)	26/170 (15)	29/267 (11)	0.22
CIC increase (%)	36/422 (8)	17/164 (10)	19/258 (7)	0.28
IgM increase (%)	28/333 (8)	8/127 (6)	20/206 (10)	0.32
IgG increase (%)	8/333 (2)	1/128 (1)	7/205 (3)	0.16
IgA increase (%)	21/315 (7)	7/123 (6)	14/192 (7)	0.84
Lymphocyte populations				
CD4 cells				
Low	17/386 (4)	6/158 (4)	11/228 (5)	0.35
Increase	19/386 (5)	5/158 (3)	14/228 (6)	
CD8 cells				
Low	10/391 (3)	7/160 (4)	3/231 (1)	0.15
Increase	25/391 (7)	9/160 (6)	16/231 (7)	
CD16 cells increase	22/192 (11)	9/89 (10)	13/103 (13)	0.35
CD19 cells increase	21/190 (11)	7/86 (8)	14/104 (13)	0.40
CD3 cells increase	27/361 (7)	7/144 (5)	20/217 ( 9)	0.27

**Table 5:** Comparison of serum TNFα and INFγ concentrations (mean ± SD) between patients with MD and controls.

Cytokine	Bilateral (N = 29)	Unilateral (N = 44)	Controls (N = 99)	P value
TNFα	16.6±21.6	11.7±19.8	11.1±15.0	0.32
INFγ	5.0±6.5	3.7±3.2	3.5±3.5	0.25

### Predictive factors for hearing loss and vertigo

Two multiple linear regression models were designed by using hearing stage and the frequency of vertigo as the dependent variables to determine which factors could explain their variability (Table 6). Hearing stage depended upon bilaterality, hearing loss at diagnosis and lymphocyte T (CD19) cells (R^2^ = 0.52, p = 0.01). In addition, patients who reported vertigo in the last six months showed a significant association with the score in the functional scale, lymphocyte B (CD8) cells and complement factor C4 (R^2^ = 0.12, p = 0.05).

**Table 6:** Multiple linear regression analysis for clinical and immunological variables associated with hearing loss and frequency of vertigo in MD.

Symptom	Factor	R^2^	R^2^ corrected	B	p value
Hearing loss	(Constant)	0.52	0.51	0.41	0.021
N = 186	1 Bilateral hearing loss			0.47	8.6×10^−6^
	2 Hearing loss at diagnosis			0.03	1.5×10^−18^
	3 CD19 cells increase			−0.36	0.010
Vertigo	(Constant)	0.12	0.11	0.30	2.9×10^−8^
N = 365	1 Functional level			0.11	5.1×10^−10^
	2 CD8 cells increase			−0.20	0.015
	3 C4 increase			0.30	0.054

B is the coefficient of regression.

## Discussion

The frequency of vertigo attacks and the SNHL are the most remarkable features of MD and they show a significant variability among patients. We have found an increased prevalence of systemic AD such as RA, SLE and AS in our series, and an association between migraine and AD not previously reported in patients with MD.

We have preferred to use the term bilateral SNHL instead of bilateral MD, since it is difficult to know which ear is causing an episode of vertigo in cases with synchronic SNHL.

Hearing loss at diagnosis is worse in patients with bilateral SNHL and this could be explain either by a diagnostic delay of these cases or by a more severe onset with hearing loss in these patients. The profile of SNHL was different in patients with unilateral or bilateral hearing loss, and when hearing levels were corrected by age, low-tone hearing loss was found in unilateral MD, but a pantonal hearing loss was observed in bilateral cases [21]. This is a relevant finding, since a pantonal SNHL may be a predictor of bilateral affectation [21]. Hearing stage was worse for patients with bilateral SNHL when the worst ear was compared with the affected ear in individuals with unilateral MD. Since hearing loss fluctuates in the first years [2], [3], [22], and some patients can develop the disease in the second ear, we also compared clinical and immunological variables in patients with unilateral and bilateral SNHL with at least 15 years since the onset of the disease, to avoid the inclusion of bilateral cases as unilateral MD (cases which potentially can develop the disease in the second ear). The mean time course in these subsets was 21 and 22 years, but no difference were found at hearing levels, suggesting that after 20 years of the disease the mean SNHL seems to be the same in all patients.

Headache is a common complaint in these patients either with uni or bilateral hearing loss, but patients with a clinical diagnosis of migraine according to the IHS criteria was observed with the same frequency as described in the general population, confirming the findings in a recent wide epidemiologic study [23]. Our current data do not support the hypothesis that migraine is more frequent in patients with bilateral SNHL.

Previous studies have shown that the number of episodes of vertigo is greater in the first few years of the disease [2], [3], [19], and the number of patients without episodes of vertigo increases with time [19]. However, these studies did not include enough number of patients with bilateral SNHL to investigate clinical differences between uni and bilateral MD.

Our study shows differences in the number of years with episodes of vertigo between patients with unilateral and bilateral SNHL. Vertigo attacks last two more years in patients with bilateral hearing loss, suggesting that both ears could be involved in the occurrence of the spells. Previous studies defined two phases in the natural evolution of episodes of vertigo in MD: the first eight years with more crises in the initial four years, and a second phase which extend from 9 to 20 years with a stable frequency of episodes [19]. Our Kaplan-Meier analyses demonstrate that the time-course of episodes of vertigo is different in patients with unilateral and bilateral SNHL, suggesting that the condition causing the episodes may persist for a longer period of time in cases with bilateral SNHL.

Autoimmune inner ear disease (AIED) is a syndrome of rapidly progressive, often fluctuating, bilateral SNHL over a period of weeks to months. Vestibular symptoms may be present in almost 50% of patients and systemic AD coexists in 15–30% of patients [24]. In some cases, AIED begins as sudden SNHL in one ear progressing rapidly to the second ear with tinnitus and balance symptoms which may resemble MD [25]. Currently, there is not biological marker for diagnosis of AIED or MD, but immune dysfunction is increasingly being recognized in MD. Patients with AIED treated with corticosteroids may improve SNHL in 50% cases at short term and in 14% after 34 months [26], so the response to steroids is not a reliable criteria to exclude MD, according to the AAO-HNS guidelines [18]. We have found an increase of systemic AD in patients with MD in our series (RA, SLE and AS). For RA and AS, the estimated prevalence in patients with MD was 2–3 times more common [27], but for SLE the prevalence was 8 times more frequent [28]. Although small series of patients with SLE has described cases with SNHL, vertigo is not a usual symptom in these patients. A recent study described a high-frequency SNHL and abnormal findings in vestibular testing in a series of patients with AS, but this findings do not fullfill the criteria for diagnosis of MD [29].

We propose that AIED would be a continuum that would include sudden SNHL, rapidly progressive SNHL or MD that could be associated with other autoimmune conditions such as systemic AD.

Moreover, systemic ADs were most commonly observed in patients with MD and migraine than in cases with MD and tension-type headache. This finding suggests that AD should be considered in all cases, but especially in patients with MD and migraine. Further studies are needed to confirm the spectrum of MD-AD-migraine.

Our data show that an immunological panel including ANA, Igs, C3, C4, CIC, TNF and INF can not be used as biological marker for diagnosis of patients with AIED or predictor for uni or bilateral SNHL. Although several reports found elevated CIC in 54–96% of patients with MD using a polyethylene glycol precipitation assay [10], [30], another study using our method, a solid phase immunosorbent assay (ELISA) for C1q and IgG also found an elevation of CICs in 4% of 49 patients with MD [31]. Our data confirm this study and found elevated CIC in 8% of 422 patients. Moreover, subanalysis of patients with uni or bilateral MD did not show a higher frequency of elevated CIC. Our data cannot support that pathophysiology of MD is different in patients with uni or bilateral involvement, although patients with bilateral SNHL display a more severe disease.

Finally, we had shown that bilaterality and SNHL at diagnosis are stronger predictors of hearing levels during follow-up. Moreover, lymphocytes B are associated with the hearing level and CD8+ T lymphocytes are associated with the recurrence of vertigo, suggesting that the immune response may determine the outcome of MD. CD4+ T lymphocytes predominate in the endolymphatic sac, while CD8+ T lymphocytes are normally scanty, but the relationship between CD4+ and CD8+ T cells may be inverted in case of chronic antigen stimulation, as in the presence of acoustic neuroma [32]. Monitoring the CD4+ and CD8+ T lymphocytes during the spells of vertigo could help to define AIED and MD.

Despite some limitations of a case series of MD, our study has found a higher prevalence of systemic AD within patients with MD. Moreover, these patients are more likely to have migraine and they may represent an endophenotype within MD. Additional muticenter studies should confirm the high prevalence of SLE, RA and AS and the relationship with migraine.

### Conclusions

- Patients with MD display an elevated prevalence of systemic ADs such as RA, SLE and AS.
- AD was more frequent in patients with MD and migraine, despite the prevelance of migraine in MD in our series does not differ from the observed prevalence in the general population.
- B lymphocytes are related with hearing loss and and CD8+ T lymphocytes with persistence of vertigo, suggesting a role for the immune response in the activity and progression of MD.
